# Instrumental variables in the cost of illness featuring type 2 diabetes

**DOI:** 10.1111/1475-6773.14412

**Published:** 2024-11-26

**Authors:** Kyle Kole, Cathleen D. Zick, Barbara B. Brown, David S. Curtis, Lori Kowaleski‐Jones, Huong D. Meeks, Ken R. Smith

**Affiliations:** ^1^ Department of Family and Consumer Studies University of Utah Salt Lake City Utah USA; ^2^ Department of Pediatrics University of Utah Salt Lake City Utah USA

**Keywords:** family health history, healthcare cost, instrumental variables, two‐part model, type 2 diabetes

## Abstract

**Objective:**

To ascertain how an instrumental variables (IV) model can improve upon the estimates obtained from traditional cost‐of‐illness (COI) models that treat health conditions as predetermined.

**Study Setting and Design:**

A simulation study based on observational data compares the coefficients and average marginal effects from an IV model to a traditional COI model when an unobservable confounder is introduced. The two approaches are then applied to real data, using a kinship‐weighted family history as an instrument, and differences are interpreted within the context of the findings from the simulation study.

**Data Sources and Analytic Sample:**

The case study utilizes secondary data on type 2 diabetes mellitus (T2DM) status to examine healthcare costs attributable to the disease. The data come from Utah residents born between 1950 and 1970 with medical insurance coverage whose demographic information is contained in the Utah Population Database. Those data are linked to insurance claims from Utah's All‐Payer Claims Database for the analyses.

**Principal Findings:**

The simulation confirms that estimated T2DM healthcare cost coefficients are biased when traditional COI models do not account for unobserved characteristics that influence both the risk of illness and healthcare costs. This bias can be corrected to a certain extent with instrumental variables. An IV model with a validated instrument estimates that 2014 costs for an individual age 45–64 with T2DM are 27% (95% CI: 2.9% to 51.9%) higher than those for an otherwise comparable individual who does not have T2DM.

**Conclusions:**

Researchers studying the COI for chronic diseases should assess the possibility that traditional estimates may be subject to bias because of unobserved characteristics. Doing so may be especially important for prevention and intervention studies that turn to COI studies to assess the cost savings associated with such initiatives.

**What Is Known on this Topic**

- Cost‐of‐illness (COI) studies that use large datasets typically estimate healthcare costs by implementing regression‐based methods.
- The potential for bias from unobserved characteristics is likely when implementing regression‐based methods on chronic diseases.
- Instrumental variables (IV) are frequently proposed as a way to purge a coefficient of this bias.

**What this Study Adds**

- A simulation study shows that COI studies can lead to biased estimates when endogeneity goes unaccounted for and the possible benefits of utilizing an IV model.
- An application of an IV model to healthcare costs in 2014 suggests that type 2 diabetes mellitus raises costs by 27% (95% CI: 2.9% to 51.9%).
- COI studies for chronic diseases can result in biased estimates if unobserved characteristics are not accounted for. With a suitable instrument, IV models can reduce this bias.

## 1 INTRODUCTION

Cost‐of‐illness (COI) studies provide information on the potential healthcare cost savings associated with health interventions that aim to reduce the risk of contracting a disease. A variety of methodological approaches are used in COI studies.^1^, ^2^ Researchers working with large data sets generally utilize regression‐based analyses.^3^ Yet, it is important to note that regression‐based COI studies are subject to modeling decisions that may bias their causality estimates, therefore limiting their usefulness.^4^ In this paper, we identify conditions when bias may occur because of unobserved characteristics and assess the ability of an instrumental variables (IV) model to address the bias.

The potential for bias from unobserved characteristics is especially likely when estimating chronic disease COI models. In such situations, the target disease may be correlated with other unmeasured health conditions affecting costs, or there may be systematic differences in the timing of diagnoses (e.g., because of differential access to the healthcare system) that also affect costs. For example, Cawley and Meyerhoefer^5^ argue that standard estimates of obesity‐related healthcare costs are likely biased because (1) obese individuals often have prior experiences that relate to both the risk of obesity and healthcare costs (e.g., poor diets), and (2) people who have less access to health care are more likely to develop obesity.

Standard COI regression‐based studies treat the health condition in question as unrelated to any unobserved characteristics. That is, the individual's health status is determined outside of the COI model and deemed exogenous.^6^ In contrast, in COI studies where health conditions and healthcare costs have reciprocal relationships, health economists state that the diagnosis and healthcare costs are *endogenous*. Formally, endogeneity is defined as an independent variable that is correlated with the error term of the regression equation with the dependent variable of interest.^6^ This means the independent and dependent variables influence each other or some unmeasured factor influences them both. In COI studies, the independent variable would be the incidence of the health condition, as opposed to its diagnosis, and the substantive outcome of interest would be healthcare costs.

Estimation of an IV model is frequently proposed as a way to purge a coefficient of bias that might otherwise exist when endogeneity is present.^6^, ^7^, ^8^ In the case of COI studies, the appropriateness of using an IV approach hinges on identifying a variable that is strongly associated with the health condition but unrelated to healthcare costs or any unobserved factors related to such costs. Testing for the presence of endogeneity and evaluating IV candidates involves a number of statistical steps described below. The steps are illustrated using individual‐level data on type 2 diabetes mellitus (T2DM) status and total annual healthcare costs. Diabetes is used for our case study because it is one of the costliest chronic diseases in the United States.^9^, ^10^, ^11^, ^12^

## 2 OVERVIEW OF ENDOGENEITY AND AN IV APPROACH TO COI STUDIES

### 2.1 Assessing the presence of endogeneity

Researchers undertaking regression‐based COI studies should begin by assessing the conceptual and empirical underpinnings for the argument that a diagnosis and costs may be endogenous. In some cases, there may be no conceptual or empirical reason to suspect endogeneity. For instance, standard COI studies of genetic disorders discovered through mandatory newborn screenings are less likely to suffer from endogeneity because compliance with such screening is very high.^13^ Likewise, COI studies focusing on the health costs of Huntington's disease would be less susceptible to endogeneity‐related bias because it is an inherited genetic disorder.^14^ Thus, the risk of developing Huntington's disease is not linked to other diseases that have an unmeasured lifestyle or environmental exposure component.

In contrast, the empirical evidence for endogeneity may be high in situations where the diagnosis varies by socio‐demographic characteristics, independent of incidence, and where healthcare access and costs likewise vary by such characteristics. For example, this was true in the case of obesity and its associated healthcare costs as described by Cawley and Meyerhoefer.^5^ In such instances, the potential for obtaining biased cost estimates from a standard COI regression model is substantial.

When the literature supports the possibility of endogeneity, the first step is to test for its presence using a Durbin–Wu–Hausman chi‐square test.^15^ A statistically significant chi‐square based on this test indicates the presence of endogeneity. That is, in a COI study a significant chi‐square is evidence for unobserved factors related to the diagnosis *and* healthcare costs.

### 2.2 Assessing the efficacy of an instrumental variable designed to address endogeneity

**FIGURE 1:**
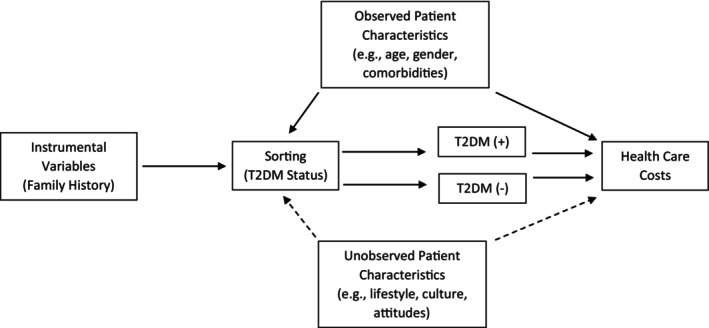
Graph of the Empirical Relationship using type 2 diabetes as a case study. The desired quantity is the healthcare costs attributable to type 2 diabetes (T2DM). In this figure, that quantity is represented by the difference between healthcare costs for T2DM positive (+) and T2DM negative (−) individuals. However, sorting into which T2DM status depends on a myriad of factors, the most troublesome of which are the unobserved characteristics. The influence of unobserved characteristics are indicated by the dashed lines and cannot be controlled for in a multivariate regression. One possible solution is to introduce an instrumental variable that affects healthcare costs only through its influence on T2DM status.

With endogeneity affirmed, the next step is to identify a variable to serve as a valid instrument capable of purging the health condition parameter estimate of statistical bias. The efficacy of the chosen instrument depends on two conditions. First, the instrument must be strongly correlated with the likelihood of the health condition's presence. Second, the instrument must affect costs only through the health condition. That is, it cannot have a direct relationship with healthcare costs nor can it be correlated with unobserved covariates that affect costs. This latter requirement is often referred to as the exclusion restriction. Figure 1 illustrates the instrument requirements of the IV method and the unbiased relationship that this method seeks to estimate.

Evaluating the strength of an instrument or set of instruments is easily done by assessing its statistical significance in the equation where the diagnosis is the dependent variable. Assuming a large sample size, an instrument is considered strong if t > 3.16 or F > 10. In an instance where there is more than one instrument, the strength of the set of instruments is assessed via a joint *F*‐test.^6^

Assessing the instrument's exclusion from the cost equation and its potential correlation with unobserved covariates affecting healthcare costs is complex because there is no definitive statistical test. However, a falsification test can be used to assess confidence in the exclusion criterion. Pizer^7^ argues that two versions of the falsification test can prove useful in evaluating if an instrument meets the exclusion criterion. In one version applied to COI estimation, the analyst would replace cost with a dependent variable that is not affected by the health condition but might be associated with potential confounders that may also be linked to the instrument. In the second version, healthcare costs remain the main outcome of interest. Estimation is done with a different sample unaffected by the health condition, but where the instrument might again be linked to potential confounders that could impact costs. Essentially, in the case of COI studies, the goal with either approach is to provide evidence that the chosen instrument is unlikely to be correlated with unmeasured factors influencing healthcare costs.

### 2.3 Introducing an instrument into a two‐part COI model

A two‐part COI model is commonly recommended because it has the ability to address the large number of records with zero values and the highly skewed distribution usually exhibited in healthcare cost data.^59^ The first equation employs a logit model to estimate whether or not any costs are incurred, represented by a binary dependent variable. The second equation is a linear regression that must address highly skewed healthcare costs. Typically, healthy people have only small amounts of healthcare costs, whereas a minority of people with serious health conditions have much higher costs. Thus, the second part usually involves making a log transformation, either through taking the log of the dependent variable or employing a generalized linear model with a gamma distribution and a log link. In the case of the IV two‐part model, IV estimation is applied to each part separately via two‐stage least squares regressions using the chosen instrument to eliminate bias for the healthcare costs estimated by the coefficient for the chronic health condition of interest.

The expected healthcare cost attributable to the health condition is estimated using the same process as calculating an average marginal effect. For each individual in the sample, a predicted probability of incurring costs is estimated from part 1 and a predicted amount of the cost is estimated from part 2. These two predictions are multiplied together to produce an expected healthcare cost for an individual given their observed characteristics. This process is performed twice: one time assuming that every individual has the health condition, and another time assuming that no one has the health condition. The difference between these two is the expected healthcare cost attributable to the condition that purges the endogeneity‐induced bias.

### 2.4 Overall assessment of the IV model

A simulation can be used to provide evidence as to whether the IV model, as conceptualized, should be preferred to the standard two‐part estimation.^16^ The simulation proceeds in three steps. First, a scenario is built where there are no unobservable confounders affecting both the risk of diagnosis and healthcare costs. The simulation then introduces an otherwise unobservable confounder to show that coefficients are biased when not accounting for this confounder. Finally, the simulation generates evidence illustrating what can happen when a valid IV is introduced to address the influence of the unobservable confounder. If the IV reduces the bias, then this is evidence that the IV model is preferred.

## 3 CASE STUDY: T2DM AND HEALTHCARE COSTS

### 3.1 Data

Data for our case study come from the Utah Population Database (UPDB),^17^ a unique research resource that contains longitudinal, individual‐level information on demographic, genealogical, residential, and medical data for all individuals living in Utah who are observed in any approved public record spanning the last 25 years. These data are linked to Utah's All‐Payer Claims Database (APCD), which contains information on healthcare costs and diagnoses for individuals who have private health insurance or Medicaid insurance.^17^ Use of the UPDB/APCD data for this study was approved by the Resource for Genetic and Epidemiologic Research, a regulatory body overseeing access to UPDB, and the University of Utah's Institutional Review Board.

Individuals represented in UPDB were initially selected based on criteria that are part of a larger intergenerational diabetes study. Specifically, individuals (1) were born between 1950 and 1970 and (2) had at least one child born in Utah between 1970 and 1990 in the four‐county urban core (i.e., Davis, Salt Lake, Utah, and Weber counties). Restricting birth years minimized the number of Medicare‐eligible individuals, whose data are not available in Utah's APCD. Requiring at least one child born in the state ensured that we have birth certificate data, which is our only source of an individual's educational attainment, race, and ethnicity.

We focus on health insurance claims made in 2014 for two reasons: first, it is the year when we observe the maximum number of individuals prior to being Medicare‐eligible given our sample definition. Individuals in our sample who became Medicare‐eligible in 2015 and enrolled would no longer be observed in the APCD data. Second, data in 2014 are more complete than subsequent years because of the 2016 Supreme Court ruling that exempted self‐funded employee health plans from state requirements to provide data to APCDs.^37^ In 2014, Utah had 37 APCD data suppliers that covered approximately 80 percent of the Utah population.^38^

There are 231,738 individuals in UPDB who met our initial sample requirements. To best capture information on healthcare costs via claims and minimize measurement error due to gaps in coverage, the sample is further limited to those having at least 9 months of medical insurance coverage in 2014 in APCD, which excludes 55,824 individuals. The inclusion of individuals is based on medical coverage rather than prescription drug coverage because the bulk of healthcare costs is attributable to medical procedures and most health insurance policies include prescription drug coverage. This follows the approach of others who have used APCD data.^18^ Altogether, there are 175,914 individuals in the final analytic sample.

### 3.2 Measures

T2DM diagnoses were based on UPDB medical information (including statewide inpatient discharge, ambulatory surgery, and emergency department records for all state‐licensed hospitals from 1996 to 2021), UPDB death certificates, and insurance claims from APCD. T2DM was defined based on the International Classification of Disease (ICD) codes, a list of which is included in the supplementary materials section S1. Individuals are considered as having T2DM in 2014 if a T2DM diagnosis had been recorded at any point up to and including 2014. To measure an individual's 2014 healthcare costs, we summed all costs paid by private insurance, public insurance, and the individual.

Data on an individual's gender, age, race/ethnicity, and educational attainment were derived from UPDB. We include a dummy variable that measures if there were 4+ months with no prescription drug coverage which captures those instances where someone had health insurance coverage for at least 9 months but not prescription drug coverage for anywhere between a third to the entirety of the year as this could have an impact on annual healthcare costs. Comorbidities are obtained from the same medical records that provide a T2DM diagnosis and were summarized by the Charlson Comorbidity Index (CCI),^19^, ^20^, ^21^ a widely‐used summary measure of major medical diagnoses that is an indicator of disease burden. Comorbidities included in this study cover a wide range of common afflictions associated with T2DM, such as renal disease, congestive heart failure, and peripheral vascular disease. Other diseases captured by the CCI include HIV, cancer, and dementia. While T2DM can be a component of the CCI, we omit T2DM from the index because a separate T2DM dummy variable is included as the main covariate of interest in our models. The numerical categories associated with this index, as measured in 2014, range from 0 (no comorbidities) to 5+ (i.e., 5 or more comorbidities excluding T2DM).

The candidate instrument is the individual's family history of T2DM. Familial T2DM histories have been used as instruments in other studies of T2DM and employment,^22^, ^23^ and our measure is in keeping with the growing recognition of the potential to use genetic markers as instrumental variables.^24^ The conceptual evidence for T2DM heritability is striking. For example, one study reported the heritability of T2DM across identical twins to be in excess of 72%.^25^ Another study found the relative risk of developing T2DM to be 2.77 for full siblings and 5.76 for twins.^26^ Moreover, the relative risk is substantially lower for spouses (who share a contemporaneous family environment but not genes) than it is for genetically related siblings.^26^, ^27^

Our weighted Family History Index (FHI) leverages genealogical and health information in UPDB to create a single index combining the number of first‐, second‐, and third‐degree relatives observed to have T2DM by 2014. Each affected individual is weighted by the strength of their genetic relatedness, via a kinship coefficient. For example, a biological parent with T2DM carries a weight of 0.50 while a first‐cousin diagnosed with T2DM carries a weight of 0.125. More details about the FHI construction can be found in the supplemental materials.

Table 1 provides descriptive information for the variables using the UPDB/APCD sample, including bivariate tests for mean differences by T2DM status. In 2014, 13.73% of individuals in this sample had been diagnosed with T2DM. Bivariate comparisons reveal mean healthcare costs for individuals with T2DM were $14,512 while mean costs for individuals without T2DM were $6054. The average difference of $8458, is comparable to the national 2017 marginal healthcare cost estimates for individuals with diabetes.^11^

**TABLE 1:** Descriptive statistics.

	Overall	By T2DM Status		
Characteristic	*N* = 175,914^a^	T2DM(−), *N* = 151,768^a^	T2DM(+), *N* = 24,146^a^	*p*‐value^b^
Indicator for Cost				<0.001
Cost = 0	13,664 (7.8%)	13,326 (8.8%)	338 (1.4%)	
Cost >0	162,250 (92%)	138,442 (91%)	23,808 (99%)	
Amount of Cost	7214.66 (20,945.71)	6053.68 (18,028.81)	14,511.93 (33,039.35)	<0.001
Age as of 2014	55.58 (5.45)	55.34 (5.47)	57.08 (5.08)	<0.001
Sex				<0.001
Female	98,569 (56%)	86,303 (57%)	12,266 (51%)	
Male	77,345 (44%)	65,465 (43%)	11,880 (49%)	
# of Children	3.33 (1.60)	3.34 (1.60)	3.27 (1.59)	<0.001
# of Eligible Relatives	3.19 (2.73)	3.21 (2.73)	3.03 (2.69)	<0.001
# of FDR w/ T2DM	0.79 (1.12)	0.73 (1.05)	1.13 (1.41)	<0.001
Ethnicity				<0.001
Non‐Hispanic White	157,987 (90%)	137,436 (91%)	20,551 (85%)	
Hispanic	12,708 (7.2%)	10,210 (6.7%)	2498 (10%)	
Other or Unknown	5219 (3.0%)	4122 (2.7%)	1097 (4.5%)	
Maximum Education Level				<0.001
High School Degree	53,691 (31%)	45,565 (30%)	8126 (34%)	
Some High School	8382 (4.8%)	6628 (4.4%)	1754 (7.3%)	
Some College	60,455 (34%)	52,336 (34%)	8119 (34%)	
College Degree	28,035 (16%)	24,777 (16%)	3258 (13%)	
Post College	25,085 (14%)	22,251 (15%)	2834 (12%)	
Unknown	266 (0.2%)	211 (0.1%)	55 (0.2%)	
Months Missing MD Insurance				0.696
0	165,787 (94%)	143,041 (94%)	22,746 (94%)	
1	4107 (2.3%)	3548 (2.3%)	559 (2.3%)	
2	3063 (1.7%)	2649 (1.7%)	414 (1.7%)	
3	2957 (1.7%)	2530 (1.7%)	427 (1.8%)	
Rx Insurance				0.532
Missing 4+ mos. Rx Coverage	14,818 (8.4%)	12,759 (8.4%)	2059 (8.5%)	
Has 1–3 mos. Rx Coverage	161,096 (92%)	139,009 (92%)	22,087 (91%)	
Medicaid Enrollment				<0.001
Enrolled for 1+ Months	166,546 (95%)	145,173 (96%)	21,373 (89%)	
Never Enrolled	9368 (5.3%)	6595 (4.3%)	2773 (11%)	
Maximum CCI Excluding Diabetes				<0.001
0	113,379 (64%)	102,715 (68%)	10,664 (44%)	
1	34,851 (20%)	28,981 (19%)	5870 (24%)	
2	14,825 (8.4%)	11,512 (7.6%)	3313 (14%)	
3	5962 (3.4%)	4248 (2.8%)	1714 (7.1%)	
4	3196 (1.8%)	2173 (1.4%)	1023 (4.2%)	
5+	3701 (2.1%)	2139 (1.4%)	1562 (6.5%)	

Abbreviations: CCI, Charlson Comorbidity Index; FDR, first‐degree relatives; MD, Medical; Rx, Prescription.

^a^ *n* (%); Mean (SD).

^b^ Pearson's Chi‐squared test; Wilcoxon rank sum test comparing descriptive statistics by T2DM status.

### 3.3 Arguments for endogeneity and empirical evidence

Several features of T2DM make it likely that it is endogenous with medical costs. For example, two studies^28^, ^29^ have reported significant increases in an individual's healthcare costs several years prior to receiving a diabetes diagnosis, suggesting that either undiagnosed prediabetes/diabetes was already contributing to higher healthcare costs or that other health conditions accounted for the higher costs while also increasing the risk of diabetes.

The potential for underestimating diabetes‐related healthcare costs also exists simply because healthcare costs attributable to T2DM in undiagnosed individuals go unnoticed. One estimate is that 11.6% of adults have diabetes but approximately 23% of that 11.6% are undiagnosed.^30^ Another study indicates that while diagnosed diabetes rates have increased over time, undiagnosed rates have remained relatively constant so that in 2020 undiagnosed cases accounted for 10% of all diabetes cases.^31^

Finally, underestimating diabetes‐related healthcare costs can be a result of non‐random selection regarding diabetes diagnoses. Undiagnosed individuals have higher health risks, relative to diagnosed individuals, that may influence healthcare costs. In particular, rates of undiagnosed diabetes are higher among individuals who are older, obese, uninsured, and members of some racial/ethnic minorities.^31^, ^32^, ^33^, ^34^, ^35^ Such biases in diabetes diagnoses and the existence of at‐risk groups for T2DM could lead to an underestimation bias because the known diagnoses are possibly more representative of a subpopulation with diabetes that has lower healthcare costs compared to the rest of the population with diabetes.

Taken together, the literature suggests that the likelihood of endogeneity between medical costs and T2DM diagnoses is high. To assess this possibility in our data, we calculated the Durbin–Wu–Hausman F‐statistic for both the part 1 logistic regression and the part 2 cost equation. The associated chi‐square statistics are 369 and 17.09, respectively. Both indicate that we should reject the null hypothesis that T2DM and healthcare costs are exogenous (*p* < 0.01).

### 3.4 Assessing the instrument's validity

While the statistical threshold for assessing the strength of a proposed instrument is quite high, the weighted FHI easily exceeds the traditional threshold of *F* ≥ 10. Indeed, the weighted FHI *F* = 1527, indicating that the instrument is very strong.

We assess the evidence for our instrument meeting the exclusion criterion by undertaking a falsification test. Given limited alternative outcomes in our data, our falsification test centers on examining the relationship for a subsample of individuals in our study population *who do not have T2DM in 2014*, but who are known to contract T2DM sometime between 2015 and 2021. Table 2 presents the estimated coefficients for the falsification test using our weighted FHI instrument. For both the equation that estimates the probability of incurring healthcare costs and the healthcare costs equation, the coefficient is statistically insignificant, suggesting that our instrument is not capturing unmeasured factors that affect either parts 1 or 2 of our 2014 COI model. That is, among individuals who are later diagnosed with T2DM, a family history of T2DM does not predict whether someone has healthcare costs or the level of costs in 2014. Robustness checks with alternative measures of family T2DM history, available in the supplemental materials (Tables S1 and S2), also have statistically insignificant coefficients.

**TABLE 2:** Instrument Falsification Tests Using the Weighted FHI and the Subsample of Individuals Who are Diagnosed with T2DM Between 2015 and 2021.

	Part 1 Logit Equation	Part 2 Cost Equation
Weighted FHI Coefficient	−0.385	−0.24
Standard Error	2.56	0.98
Number of Observations	13,662	12,813

*Note*: Regressions control for age, gender, number of children, ethnicity, education, number of comorbidities excluding T2DM, number of months missing medical insurance, if missing more than 4 months of drug coverage, and if there is at least 1 month of enrollment in Medicaid.

Abbreviation: FHI, family history index.

* *p* < 0.05.

** *p* < 0.01.

### 3.5 Assessing the IV method

In light of the evidence suggesting endogeneity, we next construct a simulation to assess the performance of the proposed IV COI method in comparison to the standard two‐part regressions. In each of the three scenarios, the simulation posits that for any individual, the researcher can observe age, gender, T2DM status, the number of the individual's parents with T2DM, whether this individual has any healthcare costs, and if so, how much is spent. Random samples are constructed to reflect features of our observed data. Namely, T2DM status for the individual is modeled such that age and gender influence the likelihood of T2DM as observed in the UDPB data while maintaining the prevalence rate in the data. We assume that if a parent has T2DM, then the odds ratio for the individual to develop T2DM based on past research.^36^ T2DM status for parents is sampled from a binomial distribution with probability equal to the prevalence of T2DM in our data. Parents constitute the instrument in this simulation because they are related to the T2DM status of the individual, but are independent from anything else in the model.

The non‐zero cost decision is assumed to follow a binomial distribution with probability that depends on the individual's covariates. Similarly, the cost amount is sampled from a gamma distribution such that the mean is similar to the observed mean in the data. We remain agnostic about how the level of cost is truly distributed by also including a scenario in which cost is sampled from a lognormal distribution. The unobserved confounder is randomly generated to influence T2DM status, the decision to incur a healthcare costs, and the amount of the cost. Each of the three scenarios created for the simulation has a sample size of 100,000 and is conducted 1000 times. Supplemental materials section S3 contains more technical details regarding variable operationalization.

Table 3 reports the coefficients on T2DM for the different simulation scenarios. Under the base scenario that does not include unobserved confounders, both parts of the two‐part model estimate the coefficient on T2DM with very little bias irrespective of model specification or how cost amounts are sampled. As expected, introducing endogeneity in the following scenario leads to bias in the estimated coefficients ranging in magnitude from 0.1 for β to 0.27 for δ. After applying IV methods to address the unobserved confounder, results differ depending on model specification, but not by how cost amounts are sampled. The coefficient in part 1 of the model, examining the propensity to incur a cost in the healthcare system, is biased downwards by −0.7. For part 2, the model leads to biased coefficients when the model is specified as a GLM with a log link. The coefficient on T2DM is unbiased in Part 2 only when the model is specified as a log‐linear model and this holds regardless of the distribution generating the cost amounts.

**TABLE 3:** T2DM Simulated Coefficients Obtained from Various Model Specifications.

Simulated Distribution for Cost	Lognormal				Gamma			
	True Value	Mean (SD) of Estimates	Bias	RMSE	True Value	Mean (SD) of Estimates	Bias	RMSE
No endogeneity								
δ	1.6094	1.6112 (0.0629)	0.0017	0.0629	1.6094	1.6112 (0.0629)	0.0017	0.0629
$\beta_{\mathrm{LOG}}$	0.6931	0.6932 (0.0149)	0.0000	0.0149	0.6931	0.6942 (0.0303)	0.0011	0.0303
$\beta_{\mathrm{NLL}}$	0.6931	0.6941 (0.0377)	0.0009	0.0377	0.6931	0.6932 (0.0166)	0.0001	0.0166
$\beta_{\mathrm{GLL}}$	0.6931	0.6937 (0.036)	0.0006	0.036	0.6931	0.693 (0.016)	−0.0002	0.016
With endogeneity								
δ	1.6094	1.8788 (0.0672)	0.2694	0.2776	1.6094	1.8788 (0.0672)	0.2694	0.2776
$\beta_{\mathrm{LOG}}$	0.6931	0.7916 (0.0124)	0.0984	0.0992	0.6931	0.7907 (0.0252)	0.0976	0.1008
$\beta_{\mathrm{NLL}}$	0.6931	0.7888 (0.033)	0.0956	0.1012	0.6931	0.7876 (0.014)	0.0945	0.0955
$\beta_{\mathrm{GLL}}$	0.6931	0.7875 (0.0312)	0.0944	0.0994	0.6931	0.787 (0.0136)	0.0939	0.0949
IV model to address endogeneity								
δ	1.6094	0.9105 (0.2396)	−0.6989	0.7388	1.6094	0.9105 (0.2396)	−0.6989	0.7388
$\beta_{\mathrm{LOG}}$	0.6931	0.6916 (0.0877)	−0.0015	0.0877	0.6931	0.6863 (0.1756)	−0.0069	0.1756
$\beta_{\mathrm{NLL}}$	0.6931	0.8034 (0.2493)	0.1102	0.2725	0.6931	0.7953 (0.1117)	0.1021	0.1513
$\beta_{\mathrm{GLL}}$	0.6931	0.7823 (0.2161)	0.0892	0.2337	0.6931	0.7787 (0.1035)	0.0856	0.1343

*Note*: Results from a simulation of coefficients in a two‐part model under various scenarios. The simulation assumes two plausible distributions for healthcare cost: the lognormal and the gamma distribution. Regardless of the distribution for healthcare cost, the decision to spend is assumed to follow a logit model. δ is the coefficient on T2DM from the logistic model in Part 1 of the two‐part model for the decision to incur a cost. β is the coefficient on T2DM from the linear model in Part 2 for the amount of cost, given that costs have occurred. The estimate of β also depends on assumptions for model specification: LOG assumes an OLS model with logged costs, NLL assumes a GLM model with errors that are normally distributed and a log link function, and GLL assumes a GLM model with errors that are gamma distributed and a log link function. The simulation highlights how coefficients are unbiased with small root mean squared error (RMSE) in the no endogeneity scenario. Introducing endogeneity into the simulation creates biased coefficients with larger RMSE. Finally, applying an IV model to address the endogeneity generally attenuates bias and RMSE in the OLS model with logged costs relative to other model specifications. However, in line with the literature, bias is still present in the logistic model even after introducing an instrument.

These results align with expectations from the literature: two‐stage least squares estimation is known to result in biased coefficients when the specification uses a log or logistic link function.^37^, ^38^ The conclusion here is that the log‐linear model robustly estimates an unbiased coefficient. Because the focus is on the cost of a chronic disease, we conclude that proceeding with a log‐linear model is the best course of action. Furthermore, while the simulated instrument represents a case of perfect information about relatives' current conditions, there is still strong evidence the proposed instrument is valid despite possibly being an imperfect measure of relatives' current conditions.

### 3.6 Case study results

Given evidence that our weighted FHI is a valid instrument, we next estimate the two‐part model. The first part is a logistic regression and the second part is a log‐linear model. We compare the results from the IV approach to those obtained when a standard two‐part model is used. While the complete sets of model estimates can be found in the supplemental materials section S4, we present the coefficients associated with T2DM in Table 4. Estimation of the standard two‐part model yields T2DM coefficients of 0.059 and 0.789 for part 1 and part 2, respectively. In contrast, the IV two‐part model yields T2DM coefficients of 2.568 and 1.273 for parts 1 and 2, suggesting that the bias in the standard model translates to an underestimation of T2DM healthcare costs. Given any spending occurs, the coefficient in Part 2 of the IV model indicates that T2DM raises healthcare costs by 27%.

**TABLE 4:** Coefficient Estimates for Healthcare Costs Attributable to T2DM.

	Basic Two‐Part Model		Instrumental Variables Two‐Part Model	
	Part 1	Part 2	Part 1	Part 2
T2DM Coefficients^a^	0.059***	0.789***	2.550***	1.274***
Standard Errors	(0.002)	(0.011)	(0.750)	(0.125)

^a^ Estimating equations control for age, gender, number of children, ethnicity, education, months of missing health insurance and prescription drug insurance, comorbidities, and Medicaid status. See supplemental materials section S4 for coefficients corresponding to the controls.

*** *p* < 0.001.

The simulation study shows that while IV methods can correct for biases in the coefficient of interest, it cannot correct for biases in the marginal effects. That said, in our supplemental materials (Table S4), we report the marginal effects to be consistently higher when using the IV method compared to the standard two‐part model. The higher magnitudes of the marginal effects are robust to variations in the instrument used and the exclusion of key covariates. Exploring the direction and magnitude of the bias is beyond the scope of this paper but it is an important methodological topic for future research.

## 4 DISCUSSION

COI studies are often used to assess the benefits of interventions that seek to reduce the risk of illness or manage a chronic condition. In regression‐based COI analyses, the individual's health condition is typically assumed to be an exogenous variable in the cost equation. In reality, this assumption may be violated, especially in instances where the health condition is one where its diagnosis is related to unmeasured factors that also impact healthcare costs. If the variable is indeed endogenous, then standard two‐part cost model will yield biased coefficients. Our simulation study is evidence that such bias can be introduced via endogeneity.

Adapting an IV model can reduce such bias, but the success of the IV approach depends on identifying one or more candidates for the instrument that meet the criteria of being (1) strongly related to the likelihood of the health condition and (2) unrelated to both healthcare costs and any unmeasured factors contributing to the likelihood of having the condition and healthcare costs. In this paper, we have unique data that allowed for a quasi‐experiment based on familial history to be linked to healthcare claims. Family history is often discussed upon visiting a physician, suggesting that familial history data exist even if it is not readily linked to the individual's claims. Nevertheless, a few studies have moved away from quasi‐experimental methods by exploiting genetic variation. These Mendelian randomization studies sought to determine healthcare costs attributable to BMI, weight, or adiposity.^39^, ^40^, ^41^ There is potential for using these data to further study the healthcare costs of T2DM, but a major caveat is the context of their data: costs are not from the US healthcare system, and there are possible selection concerns with participants who readily provide genetic information.

More generally, one cannot ever be completely sure about the relationship between health conditions and health costs when using observational data. And, since the vast majority of COI studies use observational data, we believe it is important that such studies begin by reviewing the conceptual and empirical literature with an eye toward determining whether the health condition in question may or may not be endogenous with costs. If the literature suggests that such endogeneity exists, then the study should move forward with testing for it.

When the presence of endogeneity has been demonstrated, an IV approach has the potential to correct partially or fully for estimation bias. Key to utilizing IV estimation to reduce bias is identifying a valid instrument. In some instances, no suitable candidate may exist, and as a consequence, the researcher can only acknowledge that the estimates may be biased and possibly in which direction. Where there is a heritability component to the health condition in question, our case study shows that family health history may provide an appropriate candidate.

Our case study of T2DM healthcare costs illustrates the importance of beginning a COI study with an assessment of the conceptual and empirical evidence for endogeneity. Based on our review, we conclude that endogeneity likely exists and that past regression‐based COI studies that treated T2DM status as an exogenous variable^42^, ^43^, ^44^ have probably underestimated the true costs. We were then able to correct for the endogeneity in healthcare costs attributable to T2DM to a limited degree but not able to study the direction of the bias to be expected in every scenario or its extent. Quantifying the bias in healthcare costs attributable to T2DM, or any other chronic disease, is a potential avenue for future research.

## FUNDING INFORMATION

This work was conducted by the Energy Balance Research Group at the University of Utah and supported by NIDDK grants R01DK118405 and R21DK080406.

## CONFLICT OF INTEREST STATEMENT

None.

## Supporting information


Data S1.
