# Supplementary material for: Instrumental variables in the cost of illness featuring type 2 diabetes
Source: Health Serv Res. 2024 Nov 26;60(3):e14412. doi: 10.1111/1475-6773.14412 (PMC12120511; doi:10.1111/1475-6773.14412)
Supplement: Supplementary file 1 — Data S1. [file HESR-60-0-s001.docx]

**SUPPLEMENTAL MATERIALS**

**S1 Family History Index Calculation**

We define each individual in our data set to be an “ego.” For each ego we identified the following:

1. All adult first-degree relatives (i.e., parents and siblings) who were observed in UPDB at some point between 1996 and 2019. These individuals share approximately 50% of their genes with ego.
2. All adult second-degree relatives (i.e., grandparents, half-siblings, aunts, uncles, nieces, and nephews) who were observed in UPDB at some point between 1996 and 2019. These individuals share approximately 25% of their genes with ego.
3. All adult third-degree relatives (i.e., first-cousins) who were observed in UPDB at some point between 1996 and 2019. These individuals share approximately 12.5% of their genes with ego.

Health care data has been linked to all individuals in UPDB from 1996 to 2019. As a consequence, the 1996-2019 timespan is our window for observing T2DM status for ego’s relatives. For each relative, we define “Years at Risk” to be the number of years between 1996 and 2019 that a relative is observed in UPDB.

A relative is identified as being diagnosed with T2DM using the same criteria that is described for ego in the paper. Specifically, T2DM status was identified from medical records within UPDB (including statewide inpatient discharge, ambulatory surgery, and emergency department records from 1996-2019), death certificates, and Utah’s All Payers Claims Data (APCD) — all UPDB data approved to be used in this study. T2DM was defined based on the appropriate International Classification of Disease (ICD) codes, version 9 and 10 (i.e., ICD 9 codes 250, 250.02, 250.10, 250.12, 250.20, 250.22, 250.30, 250.32, 250.40, 250.42, 250.50, 250.52, 250.60, 250.62, 250.70, 250.72, 250.80, 250.82, 250.90, and 250.92 and ICD 10 codes E08.xx and E11.xx). Individuals were coded as having T2DM if any T2DM diagnosis had been recorded in the preceding data sources.

Kinship coefficients were assigned to each relative based on the fraction of the genes the person shared with ego. For instance, if one of ego’s parents had been diagnosed with T2DM, the coefficient would be 0.50, and if one of ego’s first-cousins had been diagnosed with T2DM, the kinship coefficient would be 0.125.

With the above information in hand, the following instrumental variable options were created and used in the analyses where “affected” means a relative was diagnosed with T2DM and FHI refers to Family Health Index:

1. Unweighted (by kinship coefficients) variables:
   1. Using First-Degree Relatives Only $FHI\_FDR= \frac{\sum Affected FDR}{\sum FDR's Years at Risk}$
   2. Using All Relatives:

$$Crude FHI= \frac{\sum Affected Relatives}{\sum Relative^{'}s Years at Risk}$$

2. Weighted (by kinship coefficients) variables:
   1. Using First-Degree Relatives Only:
      $Weighted FHI\_FDR= \frac{\sum Affected Relative*Associated Kinship Coefficients}{\sum Relative^{'}s Years at Risk*Associated Kinship Coefficient}$
   2. Using All Relatives:

$$Weighted FHI= \frac{\sum Affected Relative*Associated Kinship Coefficients}{\sum Relative^{'}s Years at Risk*Associated Kinship Coefficient}$$

**S2 Falsification Tests using Alternative Measures of FHI**

The falsification test presented in the main paper applies the empirical model to an alternative sample that is not affected by T2DM in the timeframe of the study. Conceptually, the exclusion restriction in this case study requires that family history affects health care costs only through T2DM status and not through unobserved confounders. The alternative sample consists of individuals who are known to contract T2DM in the future but have yet to be diagnosed with T2DM in the timeframe of the study. One advantage to examining this alternative sample is the likelihood that these individuals carry many of the same unobserved characteristics that influence T2DM status relative to the individuals with positive T2DM status. The following two tables show that for the instrument used in the main paper and associated variations, there is no significant relationship with either the likelihood of incurring health care costs or the amount incurred.

**Table S1: Falsification test for part 1 of the two-part model**

|  | | | | |
| --- | --- | --- | --- | --- |
|  | | | | |
|  | Sample of Eventually-T2DM | | | |
|  |  | | | |
|  | Weighted FHI | Unweighted FHI | FDR FHI | Count FDR |
|  | (1) | (2) | (3) | (4) |
|  | | | | |
| IV | -0.259 | 0.040 | -0.391 | -0.025 |
|  | (2.663) | (2.869) | (1.978) | (0.037) |
|  |  |  |  |  |
|  | | | | |
| Observations | 13,662 | 13,662 | 13,662 | 13,662 |
|  | | | | |
| *Note:* | ^*^p^**^p^***^p<0.01 | | | |
|  | FHI: Family History Index; FDR: First-Degree Relative | | | |

**Table S2: Falsification test for part 2 of the two-part model**

|  | | | | |
| --- | --- | --- | --- | --- |
|  | | | | |
|  | Sample of Eventually-T2DM | | | |
|  |  | | | |
|  | Weighted FHI | Unweighted FHI | FDR FHI | Count FDR |
|  | (1) | (2) | (3) | (4) |
|  | | | | |
| IV | -0.077 | -0.001 | 0.080 | -0.012 |
|  | (0.988) | (1.005) | (0.734) | (0.014) |
|  |  |  |  |  |
|  | | | | |
| Observations | 12,813 | 12,813 | 12,813 | 12,813 |
|  | | | | |
| *Note:* | ^*^p^**^p^***^p<0.01 | | | |
|  | FHI: Family History Index; FDR: First-Degree Relative | | | |

**S3 Construction of Variables in Simulation**

A base case scenario was simulated to ensure that the simulation procedure performed as expected. Specifically, before turning to the two-part model, the base case scenario attempts to simulate a logistic model and a generalized linear model (GLM) to show that the estimation procedure properly samples from the desired distribution and yields the expected coefficients.

The empirical model is assumed to have three independent covariates: age, gender, and T2DM status. Age is sampled from a uniform distribution with a minimum of 44 and a maximum of 64. Gender and T2DM status are both simulated from a binomial distribution with probabilities 0.5 and 0.1373, respectively. The chances of testing positive for T2DM was taken from UPDB to best mimic what is observed in the study.

The first dependent variable is a binary indicator for whether an individual spends on health care. This variable is simulated from a binomial distribution with probability modeled as $plogis\left( \delta_{0}+\delta_{1}age+\delta_{2}gender+\delta_{3}T2DM \right)$, where *plogis* gives the inverse logit function, and the coefficients for $\delta_{0} ,\delta_{1} ,\delta_{2}$ are obtained from a corresponding logistic regression using the data to best mimic what is observed in the study. It is assumed that $\delta_{3}=1.609$, or that T2DM raises the odds of incurring healthcare costs by a factor of 5.

The second dependent variable, reflecting how much is spent on health care, is generated from two different distributions to address sensitivity issues related to the choice of distribution. One set is generated from a lognormal distribution such that the mean of the underlying normal distribution is modeled as $\mu=\beta_{0}+\beta_{1}age+\beta_{2}gender+\beta_{3}T2DM$ and the corresponding standard deviation is the observed standard deviation for the logged amounts in the data. Another set is generated from a gamma distribution such that the mean is $\mu=e^{\beta_{0}+\beta_{1}age+\beta_{2}gender+\beta_{3}T2DM}$ and the shape parameter is presumed to be 0.3 to best match the shape of the data. The estimated shape parameter, reported by a GLM estimation, from the data is smaller. However, R experiences issues with sampling and estimation for cases in which the shape parameter is less than 0.3. Hence for practical reasons, the shape parameter is set at the chosen level. $\beta_{0} ,\beta_{1} ,\beta_{2}$ are determined using a GLM with a gamma distribution using a log link on the data. $\beta_{3}$ is presumed to be 0.6931, or that T2DM raises costs by a factor of 2.

Below is a summary of various distribution characteristics for the 1,000 generated samples in the base case. The first row is the sampled cost indicator, the second is the sampled lognormal distribution, and the third is the sampled gamma distribution:

**Table S3: Summary Statistics for the 1,000 generated samples in the base case scenario of the simulation**

| Sampled Cost Indicator |  |  | p = 0.923  (0.0009) |  |  |  |
| --- | --- | --- | --- | --- | --- | --- |
| Lognormal Distr. | Min.   5.03  (2) | 1st Qu.   2544.78  (19) | Median   8059.88  (52) | Mean   35033.52  (478) | 3rd Qu.   25564.25  (178) | Max.   17289524.83  (11537429) |
| Gamma Distr. | Min.   0  (0) | 1st Qu.   183.24  (3) | Median   1947.95  (21) | Mean   8466.94  (52) | 3rd Qu.   9228.41  (76) | Max.   405866.86  (69177) |

The typical sample has a 0.923 probability (observed rate is 0.9223) of incurring health care costs, and the amounts in the gamma distribution has a mean of 8,466 USD (observed mean is 7,215 USD). These moments look similar to what is observed in the data. The minimum of the gamma distribution is a rounding error: it’s actually about $6\times{10}^{-6}$. The mean of the lognormal distribution uses the same coefficients as the gamma distribution, which will likely not reflect the observed data, but the results remain illustrative.

Below in Figure S1 is a density of the first 50 samples of costs from a gamma distribution compared to the observed density in the data for visual comparison:

**Figure S1: A comparison of the density of costs from simulated data to that from observed data**


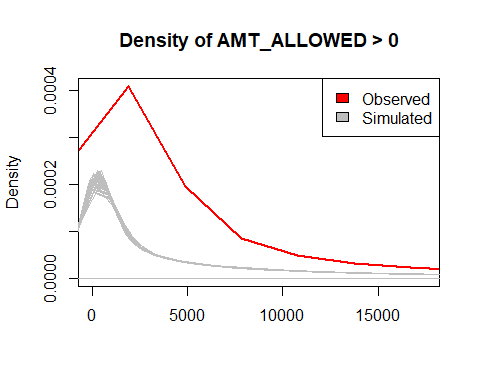


The 3 scenarios reported in the main paper sequentially builds on the base case scenario and on each other: a simulation of the base case with an additional “parents” variable, a simulation of the previous scenario now with an additional “lifestyle” variable, and a final simulation of the instrumental variables model using parents as the instrument.

The parents’ variable counts the number of parents that is observed to have T2DM. Parents are assumed to contract T2DM independently at the same rate as the observed rate in the data. This variable is sampled from a binomial distribution that is independent of any other covariate. Parents are assumed to only affect the chances of contracting T2DM for the individual.

Similarly, the lifestyle variable is sampled from the binomial distribution with a probability of 0.5. Lifestyle is posited to be independent of any other covariate in the model and is meant to reflect some unobserved characteristic that raises the chances of contracting T2DM, raises the chances of contacting the health care system, and raises the costs in the health care system. The latter two dependent variables assume that there is an independent effect of lifestyle, so that the lifestyle effect is in addition to the effect of T2DM. Having lifestyle in the model yet omitting it from the empirical model simulates the endogeneity problem.

**S4 All Coefficients from Estimating a Two-Part Model with and without Instrumental Variables**

|  | | | | |
| --- | --- | --- | --- | --- |
|  | | | | |
|  | Two-Part Models | | | |
|  |  | | | |
|  | Non-IV Pt.1 | Non-IV Pt.2 | IV Pt.1 | IV Pt.2 |
|  | (1) | (2) | (3) | (4) |
|  | | | | |
| Age | 0.002^***^ | 0.015^***^ | 0.012^***^ | 0.012^***^ |
|  | (0.0001) | (0.001) | (0.005) | (0.001) |
|  |  |  |  |  |
| # Children | -0.004^***^ | -0.031^***^ | -0.057^***^ | -0.031^***^ |
|  | (0.0004) | (0.002) | (0.006) | (0.003) |
|  |  |  |  |  |
| Male | -0.051^***^ | -0.301^***^ | -0.799^***^ | -0.323^***^ |
|  | (0.001) | (0.008) | (0.034) | (0.010) |
|  |  |  |  |  |
| Hispanic | -0.012^***^ | -0.153^***^ | -0.258^***^ | -0.181^***^ |
|  | (0.002) | (0.015) | (0.052) | (0.017) |
|  |  |  |  |  |
| Other or Unknown Ethnicity | -0.044^***^ | -0.311^***^ | -0.657^***^ | -0.348^***^ |
|  | (0.004) | (0.023) | (0.068) | (0.025) |
|  |  |  |  |  |
| Some High School Education | -0.018^***^ | -0.063^***^ | -0.300^***^ | -0.073^***^ |
|  | (0.003) | (0.019) | (0.047) | (0.020) |
|  |  |  |  |  |
| Some College Education | 0.008^***^ | 0.020^**^ | 0.133^***^ | 0.026^***^ |
|  | (0.002) | (0.009) | (0.024) | (0.010) |
|  |  |  |  |  |
| College Education | 0.019^***^ | 0.036^***^ | 0.321^***^ | 0.052^***^ |
|  | (0.002) | (0.012) | (0.036) | (0.013) |
|  |  |  |  |  |
| Post-College Education | 0.023^***^ | 0.068^***^ | 0.389^***^ | 0.088^***^ |
|  | (0.002) | (0.013) | (0.041) | (0.014) |
|  |  |  |  |  |
| Unknown Education | -0.041^**^ | -0.202^**^ | -0.509^**^ | -0.211^**^ |
|  | (0.016) | (0.100) | (0.205) | (0.096) |
|  |  |  |  |  |
| Missing 1 mo. MD Coverage | 0.014^*^ | -0.039 | 0.153 | -0.038 |
|  | (0.008) | (0.051) | (0.107) | (0.052) |
|  |  |  |  |  |
| Missing 2 mos. MD Coverage | -0.044^***^ | -0.125^*^ | -0.401^***^ | -0.115^*^ |
|  | (0.010) | (0.064) | (0.124) | (0.068) |
|  |  |  |  |  |
| Missing 3 mos. MD Coverage | -0.048^***^ | -0.385^***^ | -0.395^***^ | -0.380^***^ |
|  | (0.010) | (0.059) | (0.111) | (0.061) |
|  |  |  |  |  |
| Missing 1 mo. Rx Coverage | -0.034^***^ | -0.111^**^ | -0.441^***^ | -0.105^*^ |
|  | (0.009) | (0.053) | (0.111) | (0.054) |
|  |  |  |  |  |
| Missing 2 mos. Rx Coverage | -0.001 | -0.107 | -0.171 | -0.111 |
|  | (0.011) | (0.066) | (0.131) | (0.068) |
|  |  |  |  |  |
| Missing 3 mos. Rx Coverage | -0.015 | 0.084 | -0.349^***^ | 0.073 |
|  | (0.009) | (0.058) | (0.114) | (0.060) |
|  |  |  |  |  |
| Missing 4+ mos. Rx Coverage | -0.099^***^ | -0.310^***^ | -1.059^***^ | -0.317^***^ |
|  | (0.002) | (0.014) | (0.027) | (0.015) |
|  |  |  |  |  |
| CCI = 1 | 0.068^***^ | 0.764^***^ | 1.110^***^ | 0.736^***^ |
|  | (0.002) | (0.010) | (0.058) | (0.012) |
|  |  |  |  |  |
| CCI = 2 | 0.073^***^ | 1.121^***^ | 1.351^***^ | 1.071^***^ |
|  | (0.002) | (0.014) | (0.100) | (0.019) |
|  |  |  |  |  |
| CCI = 3 | 0.077^***^ | 1.532^***^ | 1.713^***^ | 1.456^***^ |
|  | (0.004) | (0.021) | (0.169) | (0.029) |
|  |  |  |  |  |
| CCI = 4 | 0.075^***^ | 1.740^***^ | 1.654^***^ | 1.651^***^ |
|  | (0.005) | (0.028) | (0.217) | (0.037) |
|  |  |  |  |  |
| CCI = 5+ | 0.073^***^ | 2.385^***^ | 1.670^***^ | 2.254^***^ |
|  | (0.004) | (0.026) | (0.269) | (0.044) |
|  |  |  |  |  |
| Ever-Enrolled in Medicaid | -0.013^***^ | 0.033^*^ | -0.295^***^ | 0.082^***^ |
|  | (0.003) | (0.018) | (0.100) | (0.022) |
|  |  |  |  |  |
| T2DM(+) | 0.059^***^ | 0.789^***^ | 2.550^***^ | 1.274^***^ |
|  | (0.002) | (0.011) | (0.750) | (0.125) |
|  |  |  |  |  |
| Constant | 0.853^***^ | 6.506^***^ | 2.171^***^ | 6.578^***^ |
|  | (0.007) | (0.042) | (0.157) | (0.047) |
|  |  |  |  |  |
|  | | | | |
| Observations | 175,914 | 162,250 | 175,914 | 162,250 |
|  | | | | |
| *Note:* | ^*^p^**^p^***^p<0.01 | | | |
|  | MD: Medical; Rx: Prescription; CCI: Charlson Comorbidity Index | | | |

**S5 Robustness Checks for Marginal Effects**

The IV two-part model could be sensitive to various choices in how to model the data. Table S4 tests whether the model is sensitive to the chosen instrument. Marginal effects do not seem to vary dramatically in magnitude when implementing variations of the family history index. Table S5 tests whether the model is sensitive to the inclusion of the comorbidity index and the definition of T2DM. Similarly, marginal effects do not seem to vary dramatically in magnitude under these specifications. These robustness checks suggest that the results in the main paper are robust against changes in the construction of the instrument, definition of T2DM status, and the choice of covariates.

**Table S4: Marginal effects from a two-part model using variations of the family history index**

|  | Weighted FHI | Unweighted FHI | FDR FHI | Count of FDR |
| --- | --- | --- | --- | --- |
| Overall Sample | $5,112  (769) | $5,534  (946) | $4,714  (538) | $4,858  (425) |
| Sample with Diabetes | $7,451  (1055) | $8,029  (1291) | $6,903  (745) | $7,102  (585) |
| Bootstrapped standard errors using 1,000 replications in parentheses | | | | |
| FHI: Family History Index; FDR: First-Degree Relatives | | | | |

**Table S5: Marginal effects from various specifications of the two-part model**

|  | Omit CCI from Covariates | Alternate Diagnosis Definition |
| --- | --- | --- |
| Overall Sample | $7,991  (1102) | $6,017  (978) |
| Sample with Diabetes | $8,738  (1167) | $8,997  (1380) |
| Bootstrapped standard errors using 1,000 replications in parentheses | | |
| CCI: Charlson Comorbidity Index | | |
